# Drug Repositioning with GraphSAGE and Clustering Constraints Based on Drug and Disease Networks

**DOI:** 10.3389/fphar.2022.872785

**Published:** 2022-05-10

**Authors:** Yuchen Zhang, Xiujuan Lei, Yi Pan, Fang-Xiang Wu

**Affiliations:** ^1^ School of Computer Science, Shaanxi Normal University, Xi’an, China; ^2^ Faculty of Computer Science and Control Engineering, Shenzhen Institute of Advanced Technology, Chinese Academy of Sciences, Shenzhen, China; ^3^ Division of Biomedical Engineering, University of Saskatchewan, Saskatoon, SK, Canada

**Keywords:** drug reposition, graph neural network, GraphSAGE, matrix factorization, clustering constraint, COVID-19

## Abstract

The understanding of therapeutic properties is important in drug repositioning and drug discovery. However, chemical or clinical trials are expensive and inefficient to characterize the therapeutic properties of drugs. Recently, artificial intelligence (AI)-assisted algorithms have received extensive attention for discovering the potential therapeutic properties of drugs and speeding up drug development. In this study, we propose a new method based on GraphSAGE and clustering constraints (DRGCC) to investigate the potential therapeutic properties of drugs for drug repositioning. First, the drug structure features and disease symptom features are extracted. Second, the drug–drug interaction network and disease similarity network are constructed according to the drug–gene and disease–gene relationships. Matrix factorization is adopted to extract the clustering features of networks. Then, all the features are fed to the GraphSAGE to predict new associations between existing drugs and diseases. Benchmark comparisons on two different datasets show that our method has reliable predictive performance and outperforms other six competing. We have also conducted case studies on existing drugs and diseases and aimed to predict drugs that may be effective for the novel coronavirus disease 2019 (COVID-19). Among the predicted anti-COVID-19 drug candidates, some drugs are being clinically studied by pharmacologists, and their binding sites to COVID-19-related protein receptors have been found *via* the molecular docking technology.

## Introduction

Traditional drug discovery is often based on a specific disease. It generally has a number of stages, including target discovery, target validation, lead compound identification, lead optimization, preclinical drug development, advancing to clinical trials, and clinical trials. Typically, the development of an effective drug takes an average of 15 years and costs 800 million to 1.5 billion US dollars (Dudley et al., 2011) (Yu et al., 2015). However, the success rate is often not high due to the lack of systematic evaluation of other indications that drugs can treat, as well as the impact of our life, disease development, and market factors. These difficulties have caused pharmaceutical companies very worrisome when developing new drugs, and the development speed is slow (Booth and Zemmel, 2004).

From cheminformatics and life sciences (Bader et al., 2008), it is well acknowledged that one drug may work on multiple target proteins, and one target protein is related to multiple diseases, which is the basis of drug repositioning. Actually, drug repositioning brings significant benefits to drug research and related pharmaceutical companies. For example, minoxidil (Varothai and Bergfeld, 2014), a drug originally used to relieve hypertension and excessive tension was later found to effectively treat symptoms such as hair loss. Antifungal and antitumor drug itraconazole (ITZ) can act as a broad-spectrum enterovirus inhibitor (Strating et al., 2015). However, this kind of drug repositioning is mostly based on clinical accidental discoveries and the experience of pharmacists, and it is difficult for large-scale investigation.

With the development of cross-technology, more and more researchers tend to use computational technologies to predict new indications of existing drugs. These methods mainly include network propagation, low-rank matrix approximation, and graph neural network. Based on biological networks, similarity measures and bi-random walk were proposed for drug repositioning (Luo et al., 2016). Yu *et al.* combined miRNAs and group specificity to predict potential therapeutic drugs for breast cancer (Yu et al., 2018). A genome-wide positioning systems network algorithm was developed for drug repurposing (Cheng et al., 2019). Fiscon *et al.* presented a new network-based algorithm SAveRUNNER and applied it to COVID-19 (Fiscon et al., 2021). However, due to the complexity and noise of interactions between organisms, the prediction accuracy based on those existing methods cannot meet the requirements. Some methods were developed based on low-rank matrix approximation. Luo *et al.* proposed a drug repositioning recommendation system (DRRS) to predict novel drug indications based on low-rank matrix approximation and randomized algorithms (Luo et al., 2018). Wang *et al.* proposed a projection onto convex sets (Wang et al., 2019) to relocate the functions of drugs. Weight graph regularized matrix factorization was also used in drug response prediction (Guan et al., 2019). Wu *et al.* used meta paths and singular value decomposition to predict drug–disease associations (Wu et al., 2019). Yang *et al.* used a bounded nuclear norm regularization (BNNR) method to complete the drug–disease matrix (Yang et al., 2019). An improved drug repositioning approach using Bayesian inductive matrix completion also was proposed (Zhang W. et al., 2020). Meng *et al.* used the similarity-constrained probabilistic matrix factorization for drug repositioning and applied it to COVID-19 (Meng et al., 2021). However, these matrix-based methods did not take the biochemical properties of drugs and diseases into consideration.

With the widespread application of artificial intelligence technology, more and more machine learning and deep learning methods are also applied to drug development and other fields of bioinformatics. Regularized kernel classifier was proposed to predict new drug–disease associations (Lu and Yu, 2018). Madhukar *et al.* used a Bayesian machine learning approach to identify drug targets with diverse data types (Madhukar et al., 2019). Huang *et al.* proposed a network embedding-based method CMFMTL for predicting drug–disease associations. CMFMTL handled the problem as multi-task learning where each task is to predict one type of association, and two tasks complement and improve each other by capturing the relatedness between them (Huang et al., 2020). Zhu *et al.* constructed a drug knowledge graph for drug repurposingand transformed information in the drug knowledge graph into valuable inputs to allow machine learning models to predict drug repurposing candidates (Zhu et al., 2020). Zeng *et al.* developed a network-based deep learning approach, termed deepDR (Zeng et al., 2019), for *in silico* drug repurposing. Li *et al.* used molecular structures and clinical symptoms *via* a deep convolutional neural network to identify drug–disease associations (Li Z et al., 2019). A network embedding method called NEDD (Zhou et al., 2020) was proposed to predict novel associations between drugs and diseases using meta paths of different lengths.

Graph convolutional network (GCN) methods have also been further used in the field of medicine. A layer attention graph convolutional network (LAGCN) (Yu et al., 2020) was also used by fusing heterogeneous information to the GCN. They introduced a layer attention mechanism to combine embeddings from multiple graph convolution layers for further improving the prediction performance (Cai et al., 2021). Wang *et al.* also proposed a global graph feature learning method to predict associations (Wang et al., 2022). Meta path-based methods such as metapath2vec and meta-structure have also been developed (Zhang Y. et al., 2020; Lei et al., 2021). Algorithms based on graph neural networks (GNNs) or graph embeddings consider both biochemical characteristics and network interactions, but they often have high time complexity and do not consider the characteristic of drug clusters or combination drugs. At the same time, when extracting features of drug–disease associations, a large number of methods only directly connect drug features and disease features without considering the influence of different features. The feature representation of association needs to be improved.

**FIGURE 1:**
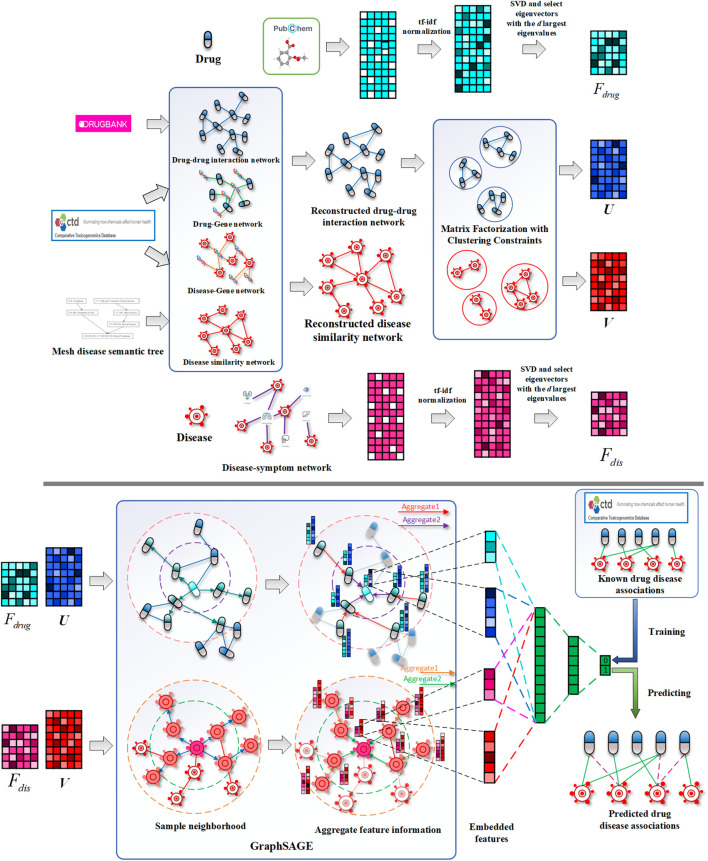
Schematic diagrams of data processing and DRGCC model. 1) Reconstruct the drug interaction network and establish the disease similarity network, 2) extract the attributes feature of drugs and diseases, 3) obtain network clustering features of drugs and diseases, and 4) construct the GraphSAGE prediction model to predict potential drug–disease relationships.

While existing methods cannot accurately predict the potential drug–disease associations, and the network is often unchangeable after model training, we proposed a drug repositioning method DRGCC based on network clustering constraints and GraphSAGE. First, we extracted the molecular structure features of drugs and the symptom features of diseases as the biological attribute features. After that, we used the associations between drugs and genes, as well as the relationships between diseases and genes, to reconstruct a drug–drug interaction network and establish a disease similarity network. The third step was to use a clustering algorithm to divide the two networks into some clusters. The network clustering features of drugs and diseases were obtained by matrix factorization with the divided cluster set as a condition constraint, respectively. Finally, we built two GraphSAGE models based on drug and disease networks and fed the attributes and clustering features of drugs and diseases to the two models, respectively, to obtain the potential treatment probability of the existing drugs for the diseases. The method was applied to the prediction of anti-COVID-19 drugs, and some case studies were conducted. The framework of the method DRGCC is shown in Figure 1. The main contributions of this work are summarized as the following two points: 1) DRGCC integrates the clustering features of networks, which can effectively improve the prediction accuracy of drug–disease associations. 2) DRGCC can embed new nodes in the existing network and predict their associations. In addition, DRGCC is complementary to existing experimental methods to enable rapid and accurate discovery of drug candidates for anti-COVID-19 and other emerging viral infectious diseases.

## Materials and Method

In this section, we introduce the database used in the study and how they were processed. The known associations of drug and disease were obtained. The drug–drug interaction network was reconstructed. The disease similarity network was calculated. Their attribute features and network features were also extracted. The purpose of our study is to predict potential associations from known drug–disease associations, which can be formulated as a classification problem. Therefore, we developed a GNN model based on GraphSAGE, which takes the obtained drug and disease attribute features and clustering features as input, and outputs the possibility of potential relationships between them.

### Known Associations of Drugs and Diseases

Known drug and disease relationship data can be obtained from the Comparative Toxicogenomics Database (CTD) (Davis et al., 2021). CTD is a publicly available database that aims to advance understanding of how environmental exposures affect human health. It provides manually curated information about drug compound–gene/protein interactions, drug compound–disease, and gene–disease relationships. We first screened 36,392 drug–disease associations marked with therapeutic relationships in CTD (version 2021.2.26). They corresponded to 6,699 drugs and 2,472 diseases. In order to make it more focused and easier to verify the method later, we extracted drugs with more than 10 disease treatment effects and diseases that are affected by more than 10 drugs. We made the corresponding PubChem Compound ID (CID) and PubChem Substance ID (SID) (Kim et al., 2021) for each drug compound. In the end, we extracted 780 drugs, 717 diseases, and 17,594 therapeutic associations. The known drug–disease association matrix is marked as *Y*, if drug *i* has a therapeutic effect on disease *j*, then $Y_{ij}$ = 1; otherwise, it is 0. In addition, we also considered the relational database of viruses and drugs, HDVD (Meng et al., 2021), which includes 34 viruses, 219 drugs, and 455 human drug–virus interactions. In the HDVD database, SARS-CoV-2, which has recently attracted much attention, is included. The statistics of the two datasets are shown in Table 1.

**TABLE 1:** Statistics of pre-processed CTD and HDVD database.

Dataset	Drugs	Diseases/viruses	Known associations	Density
CTD	780	717	17594	0.0315
HDVD	219	34	455	0.0611

### Reconstruction of Drug–Drug Interaction Network

In daily life, we have known for a long time that there are interactions between drugs and drugs. Some combinations of drugs can promote the cure of diseases. The interactions between drugs can also provide the basis for feature extraction and fusion of drugs. DrugBank (Wishart et al., 2018) provides us with a large number of drug–drug interactions (DDIs). We found 2,669,764 interactions in the database. We denote the drug–drug interaction matrix by $M_{DDI}$ . Due to the non-correspondence of IDs, only 489 of the 780 drugs were mapped to DrugBank. There are 56,439 interactions among 489 drugs. Therefore, we aimed to use other biological properties of drugs to infer possible associations between drugs. The clinical relevance of drug–drug interactions also depends on the patient’s genetic profile. Drug–drug–gene and drug–gene–gene interactions affect the therapeutic properties of drugs (Hahn and Roll, 2021). A method for calculating drug similarity using drug–gene associations was proposed by Groza et al. (2021). Inspired by these studies, we aimed to use the drug–gene relationship to complement the existing drug interactions. The CTD also provides the relationships between drug compounds and genes. We obtained 383,525 drug–gene relationships from it. They covered 768 drugs and 34,184 genes. We denote the drug–gene association matrix by $M_{drug-gene}$ ; if drug *i* has an association with gene *j*, then $M_{drug-gene_{ij}}$ = 1; otherwise, it is 0. The reconstructed drug–drug interaction (RDDI) matrix $M_{RDDI}$ is calculated as follows:

$$M_{RDDI_{ij}}=\left\{ \begin{array}{l} \;\;\;\;M_{DDI_{ij}}\;\;\;\;if\;M_{DDI_{ij}}\neq 0,\\ \frac{\left|M_{drug-gene_{i,}}\cap M_{drug-gene_{j,}}\right|}{\left|M_{drug-gene_{i,}}\cup M_{drug-gene_{j,}}\right|}\;if\;M_{DDI_{ij}}=0. \end{array}\right.$$ (1)

These associated genes often encode target proteins, and thus, we considered the relationship between drugs and target proteins, making the drug interaction network more complete.

### Construction of Disease Similarity Network

There are also similarities between diseases, and a large number of calculation methods for disease similarity have been developed in the literature. In studying the relationship between miRNAs and diseases, Cui *et al.* successively developed two versions of the method (Wang et al., 2010) (Li J. et al., 2019), both of which applied disease semantic similarity. All the denominations of diseases were in accordance with the MeSH (Yu, 2018) database (https://www.nlm.nih.gov/mesh/meshhome.html). Finally, we obtained the semantic similarity matrix $M_{DS}$ of diseases according to the method of Wang et al. (2010). Different from the method in Disease Ontology (Schriml et al., 2019) that only builds an overall semantic tree, MESH divides diseases into 17 subcategories or sub-trees, so there are null values in the calculated disease similarity for some different subcategory diseases. Previous work has shown elucidating disease and gene associations (Li et al., 2021). Similar to reconstructing the $M_{RDDI}$ , we use disease–gene relationship to reconstruct the disease similarity network. The CTD contains 13,775,363 disease–gene relationships, which cover 715 diseases and 50,827 genes. The disease–gene association matrix is denoted by $M_{dis-gene}$ . If disease *i* is related to gene *j*, then $M_{dis-gene_{ij}}$ = 1; otherwise, it is 0. The reconstructed disease similarity matrix $M_{RDS}$ is calculated as follows:

$$M_{RDS_{ij}}=\left\{ \begin{array}{l} \;\;\;\;\;\;\;M_{DS_{ij}}\;\;\;\;\;\;\;\;\;if\;M_{DS_{ij}}\neq 0,\\ \sum_{k=1}^{N_{gene}}\frac{M_{dis-gene_{ik}}M_{dis-gene_{kj}}^{T}}{\sum_{p=1}^{N_{gene}}M_{dis-gene_{ip}}\sum_{q=1}^{N_{gene}}M_{dis-gene_{qj}}^{T}}\;if\;M_{DS_{ij}}=0, \end{array}\right.$$ (2)

where $N_{gene}$ is the number of all genes.

### Processing of Attribute Features

The attribute features of drugs can be described by their structures. The PubChem system generates a binary substructure fingerprint for chemical structures. These fingerprints are used by PubChem for similarity neighboring and similarity searching (Kim et al., 2021). The structure of a drug can be described by 881 substructures, and a substructure is a fragment of a chemical structure. The fingerprint is an ordered list of binary bits (0/1). A Boolean value for each bit determines or tests the presence of a chemical structure. Binary data are stored in one-byte increments. Therefore, the length of the fingerprint is 111 bytes (888 bits), which include padding 7 bits at the end to complete the last byte. The four-byte prefix including the fingerprint bit length (881 bits) increases the size of the stored PubChem fingerprint to 115 bytes (920 bits). To learn embeddings of drugs, we also used latent semantic analysis (Deerwester et al., 1990). Let $N_{sub}$ denote the number of substructures generated from all drugs. We employ a matrix $M_{drug-sub}\in R^{N_{drug}\times N_{sub}}$ , and $M_{drug-sub}$ is defined as follows:

$$M_{drug-sub_{ij}}=tf\left(i,j\right)\cdot idf\left(N_{drug},j\right),$$ (3)

where tf(i,j) stands for the strength of the *i-th* drug having *j-th* substructure. If substructure *j* appears in drug *i*, then tf(i,j) = 1/ $N_{sub_{i}}$ ; otherwise, it is 0. $N_{sub_{i}}\;$ is the number of substructures in drug *i*.

$$idf\left(N_{drug},j\right)=\log\frac{N_{drug}}{\left|\left\{i\in drug:tf\left(i,j\right)\neq 0\right\}\right|}.$$ (4)

*idf* $\left(N_{drug},j\right)$ results in lower weights for more common substructures and higher weights for less common substructures. This is consistent with an observation in the information theory that rarer events generally have higher entropy and are thus more informative. Then, the matrix $M_{drug-sub}$ was decomposed by singular value decomposition (SVD) into three matrices R,  Σ, and Q , such that $M_{drug-sub}=R\Sigma Q$ . $\Sigma \in {\mathbb{R}}^{N_{drug}\times N_{sub}}$ is a diagonal matrix with the eigenvalues of $M_{drug-sub}$ , and *R* is an $N_{drug}\times N_{drug}$ matrix in which each column is an eigenvector $R_{.j}$ of $M_{drug-sub}$ corresponding to the eigenvalue ${\text{Σ}}_{jj}$ . Afterward, in order to embed the features into the low-dimensional space ${\mathbb{R}}^{d_{drug}}$ , we extracted the feature vectors corresponding to the top $d_{drug}$ largest singular values to form a new drug attribute feature matrix $F_{drug}$ .

Similar to drug attribute feature extraction, disease attribute features are also extracted. Diseases are often accompanied by a large number of symptoms when they occur. Zhou *et al.* established a disease–symptom network when studying the commonalities between diseases (Zhou et al., 2014). They gave 322 common symptoms for each disease, established a disease–symptom relationship matrix, and also used the term frequency-inverse document frequency method to weight. After that, we also used the SVD method to obtain a disease feature matrix $F_{dis}$ in ${\mathbb{R}}^{d_{dis}}$ space. The feature vectors corresponding to the top $d_{dis}$ largest singular values form the disease attribute feature matrix $F_{dis}$ .

### Extraction of Network Clustering Feature

In the previous section, we have obtained attribute features of drugs and diseases. However, the network features between drugs and diseases were not involved. On the other hand, numerous studies have confirmed the modularity that exists between biomolecules (Ni et al., 2020) (Groza et al., 2021). Matrix factorization, as a commonly used low-rank matrix approximation method, can achieve the goal by adding expectation constraints. Therefore, we aimed to use the matrix factorization method to measure the features of the relationship between drugs and diseases and consider the modularity of drugs and diseases. Two constraints were added to matrix factorization, one is sparsity and the other is clustering constraints. For sparsity, it is desirable to obtain a basis matrix with fewer parameters and be able to restore the original associations. It can be written as follows:

$$\min J\left(U,V\right)=\underset{U,V}{\min}\left\{\frac{1-2\alpha }{2}{\left\|P⊙\left(Y-UV\right)\right\|}_{F}^{2}+\frac{\alpha }{2}{\left\|U\right\|}_{F}^{2}+\frac{\alpha }{2}{\left\|V\right\|}_{F}^{2}\right\},$$ (5)

where $U\in R^{N_{drug}\times k}$ *,* $V\in R^{k\times N_{dis}}$ are the feature matrices of drugs and diseases, *k* can be used as the embedded feature dimension, and *P* is the observation matrix. In this matrix, the elements corresponding to positive and negative samples are marked as 1, and the other elements are 0. ⊙ is the Hadamard product. For clustering attributes, we first need to cluster nodes in the drug network and disease network. MCODE (Bader and Hogue, 2003) is a very mature network clustering method, which has been widely used in a variety of network analyses. We used it to cluster the reconstructed drug–drug interaction network and disease similarity network. When extracting features for drug and disease networks, the embedded features should satisfy the property that drugs or diseases of different clusters have greater distinguishability. Using Euclidean distance as the measure function of similarity between features, the matrix factorization subject to clustering constraints can be written as follows:

$$\min J\left(U,V\right)=\underset{U,V}{\min}\left\{\frac{1-2\alpha -2\beta }{2}{\left\|P⊙\left(Y-UV\right)\right\|}_{F}^{2}+\frac{\alpha }{2}{\left\|U\right\|}_{F}^{2}-\frac{\beta }{2}{\sum_{i=1}^{c_{\text{drug}}}\left\|{\bar{U}}^{\left(i\right)}-{\bar{U}}_{all}\right\|}_{2}^{2}+\frac{\alpha }{2}{\left\|V\right\|}_{F}^{2}-\frac{\beta }{2}{\sum_{i=1}^{c_{\text{dis}}}\left\|{\bar{V}}^{\left(i\right)}-{\bar{V}}_{all}\right\|}_{2}^{2}\right\},$$ (6)

where $c_{drug}$ and $c_{disease}$ are the cluster number of drugs and diseases, respectively; ${\bar{U}}^{\left(i\right)}\left({\bar{V}}^{\left(i\right)}\right)$ denotes the average vector of the drug (disease) feature vectors in the *i*-*th* cluster; ${\bar{U}}_{all},\left({\bar{V}}_{all}\right)$ is the average vector of all drug (disease) feature vectors; and α and β are control parameters. We set $s_{i}$ $\left(s_{i}^{'}\right)$ to the node number of *i*-*th* drug (disease) cluster, $N_{drug}=s_{1}+s_{2}+\ldots +s_{c_{drug}}$ , and $N_{dis}=s_{1}^{\prime }+s_{2}^{\prime }+\ldots +s_{c_{dis}}^{\prime }$ . To facilitate the solution, let $A_{drug}^{\left(i\right)}={\left[\frac{1}{s_{i}},\frac{1}{s_{i}},\ldots ,\frac{1}{s_{i}}\right]}_{1\times s_{i}}$ and $A_{dis}^{\left(i\right)}={\left[\frac{1}{s_{i}^{'}},\frac{1}{s_{i}^{'}},\ldots ,\frac{1}{s_{i}^{'}}\right]}_{s_{i}^{'}\times 1}^{T}$ , so the average of the feature values of *i*-*th* cluster samples can be calculated as follows:

$$\begin{array}{l} {\bar{U}}^{\left(i\right)}=A_{drug}^{\left(i\right)}{\left[U^{\left(i\right)}\left(1\right)\text{, }U^{\left(i\right)}\left(2\right)\text{, }\cdots \text{ ,}U^{\left(i\right)}\left(s_{i}\right)\right]}^{T}\\ {\bar{V}}^{\left(i\right)}=\left[V^{\left(i\right)}\left(1\right)\text{, }V^{\left(i\right)}\left(2\right)\text{, }\cdots \text{ ,}V^{\left(i\right)}\left(s_{i}^{\prime }\right)\right]A_{dis}^{\left(i\right)}, \end{array}$$ (7)

where $U^{\left(i\right)}\left(x\right)\left(V^{\left(i\right)}\left(x\right)\right)$ is the *x*-*th* feature vector of *i*-*th* drug (disease) cluster. The matrix formed by the average vector of all clusters can be represented by

$$\begin{array}{l} \bar{U}={\left[{\bar{U}}^{\left(1\right)},{\bar{U}}^{\left(2\right)},...,{\bar{U}}^{\left(c_{drug}\right)}\right]}^{T}=A_{z_{drug}}U\\ \bar{V}=\left[{\bar{V}}^{\left(1\right)},{\bar{V}}^{\left(2\right)},...,{\bar{V}}^{\left(c_{dis}\right)}\right]=VA_{z_{dis}}, \end{array}$$ (8)

where

$$A_{z_{drug}}={\left[\begin{array}{cccc} A_{dr\text{ug}}^{\left(1\right)} & & & \\ & A_{dr\text{ug}}^{\left(2\right)} & & \\ & & \ddots & \\ & & & A_{dr\text{ug}}^{\left(c_{d\text{rug}}\right)} \end{array}\right]}_{c_{d\text{rug}}\times N_{d\text{rug}}}\;A_{z_{dis}}={\left[\begin{array}{cccc} A_{dis}^{\left(1\right)} & & & \\ & A_{dis}^{\left(2\right)} & & \\ & & \ddots & \\ & & & A_{dis}^{\left(c_{dis}\right)} \end{array}\right]}_{N_{dis}\times c_{dis}}.$$ (9)

Then, we defined matrices $B_{drug}$ and $B_{disease}$ as follows:

$$B_{drug}={\left[\begin{array}{cccc} 1/N_{drug} & 1/N_{drug} & \cdots & 1/N_{drug}\\ 1/N_{drug} & 1/N_{drug} & \cdots & 1/N_{drug}\\ \vdots & \vdots & \ddots & \vdots \\ 1/N_{drug} & 1/N_{drug} & \cdots & 1/N_{drug} \end{array}\right]}_{c_{drug}\times N_{drug}}\;B_{dis}={\left[\begin{array}{cccc} 1/N_{dis} & 1/N_{dis} & \cdots & 1/N_{dis}\\ 1/N_{dis} & 1/N_{dis} & \cdots & 1/N_{dis}\\ \vdots & \vdots & \ddots & \vdots \\ 1/N_{dis} & 1/N_{dis} & \cdots & 1/N_{dis} \end{array}\right]}_{N_{dis}\times c_{dis}}.$$ (10)

${\bar{U}}_{all},\left({\bar{V}}_{all}\right)$ can be written in the following matrix form:

$$\begin{array}{l} {\left[{\bar{U}}_{all},{\bar{U}}_{all},...,{\bar{U}}_{all}\right]}^{T}c_{drug}\times k=B_{drug}U\\ {\left[{\bar{V}}_{all},{\bar{V}}_{all},...,{\bar{V}}_{all}\right]}_{k\times c_{dis}}=VB_{dis}. \end{array}$$ (11)

Therefore, the constraint term of clustering can be expressed by formula (12):

$$\begin{array}{l} {\sum_{i=1}^{c_{drug}}\left\|{\bar{U}}^{\left(i\right)}-{\bar{U}}_{all}\right\|}_{2}^{2}=tr\left(\left(A_{z_{drug}}U-B_{drug}U\right){\left(A_{z_{drug}}U-B_{drug}U\right)}^{T}\right)\\ {\sum_{i=1}^{c_{dis}}\left\|{\bar{V}}^{\left(i\right)}-{\bar{V}}_{all}\right\|}_{2}^{2}=tr\left({\left(VA_{z_{dis}}-VB_{dis}\right)}^{T}\left(VA_{z_{dis}}-VB_{dis}\right)\right). \end{array}$$ (12)

As a result, the constraint matrix factorization in formula (6) has been transformed into

$$\begin{array}{l} J\left(U,V\right)=\frac{1-2\alpha -2\beta }{2}tr\left(\left(P^{T}⊙Y^{T}\right)\left(P⊙Y\right)\right)-\left(1-2\alpha -2\beta \right)tr\left(\left(P⊙\left(UV\right)\right)\left(P^{T}⊙Y^{T}\right)\right)\\ +\frac{1-2\alpha -2\beta }{2}tr\left(P⊙\left(UV\right)\left(P^{T}⊙\left(V^{T}U^{T}\right)\right)\right)+\frac{\alpha }{2}tr\left(UU^{T}\right)+\frac{\alpha }{2}tr\left(VV^{T}\right)\\ -\frac{\beta }{2}tr\left(A_{z_{drug}}UU^{T}A_{z_{drug}}^{T}\right)+\beta tr\left(A_{z_{drug}}UU^{T}B_{drug}^{T}\right)-\frac{\beta }{2}tr\left(B_{drug}UU^{T}B_{drug}^{T}\right)\\ -\frac{\beta }{2}tr\left(A_{z_{dis}}^{T}V^{T}VA_{z_{dis}}\right)+\beta tr\left(A_{z_{dis}}^{T}V^{T}VB_{dis}\right)-\frac{\beta }{2}tr\left(B_{dis}^{T}V^{T}VB_{dis}\right). \end{array}$$ (13)

The partial derivatives of *J*(*U*, *V*) with respect to *U* and *V* are calculated as follows:

$$\begin{array}{l} \frac{\partial J\left(U,V\right)}{\partial \left(U\right)}=-\left(1-2\alpha -2\beta \right)\left(P⊙Y\right)V^{T}+\left(1-2\alpha -2\beta \right)\left(P⊙\left(UV\right)\right)V^{T}+\alpha U\\ \;\;\;\;\;\;\;-\beta A_{z_{drug}}^{T}A_{z_{drug}}U+\beta B_{drug}^{T}A_{z_{drug}}U-\beta B_{drug}^{T}B_{drug}U+\beta A_{z_{drug}}^{T}B_{drug}U,\\ \frac{\partial J\left(U,V\right)}{\partial \left(V\right)}=-\left(1-2\alpha -2\beta \right)U^{T}\left(P⊙Y\right)+\left(1-2\alpha -2\beta \right)U^{T}\left(P⊙\left(UV\right)\right)+\alpha V\\ \;\;\;\;\;\;\;-\beta VA_{z_{dis}}A_{z_{dis}}^{T}+\beta VB_{dis}A_{z_{dis}}^{T}+\beta VA_{z_{dis}}B_{dis}^{T}-\beta VB_{dis}B_{dis}^{T}. \end{array}$$ (14)

After the initial *U* and *V* are randomly given, solution is solved as per the following iterative rules until the stopping condition is met. Drug network clustering feature *U* and disease network clustering feature *V* are obtained.

$$\begin{array}{l} U_{ij}\leftarrow U_{ij}\frac{{\left(\left(1-2\alpha -2\beta \right)\left(P⊙Y\right)V^{T}+\beta A_{z_{drug}}^{T}A_{z_{drug}}U+\beta B_{drug}^{T}B_{drug}U\right)}_{ij}}{{\left(\left(1-2\alpha -2\beta \right)\left(P⊙\left(UV\right)\right)V^{T}+\alpha U+\beta \left(B_{drug}^{T}A_{z_{drug}}+A_{z_{drug}}^{T}B_{drug}\right)U\right)}_{ij}},\\ V_{ij}\leftarrow V_{ij}\frac{{\left(\left(1-2\alpha -2\beta \right)U^{T}\left(P⊙Y\right)+\beta VA_{z_{dis}}A_{z_{dis}}^{T}+\beta VB_{dis}B_{dis}^{T}\right)}_{ij}}{{\left(\left(1-2\alpha -2\beta \right)U^{T}\left(P⊙\left(UV\right)\right)+\alpha V+\beta V\left(B_{dis}A_{z_{dis}}^{T}+A_{z_{dis}}B_{dis}^{T}\right)\right)}_{ij}}. \end{array}$$ (15)

### Drug Repositioning Using GraphSAGE

GraphSAGE (SAmple and aggreGatE) (Hamilton et al., 2017) is a new graph convolutional neural (GCN) (Defferrard et al., 2016) model proposed, which has two improvements to the original GCN. On the one hand, it used the strategy of sampling neighbors to transform the GCN from a full graph training method to a node-centric small batch training method, which made large-scale data distributed training possible. On the other hand, the algorithm extended the operation of aggregating neighbors. In this study, we used the GraphSAGE model for the drug–drug interaction network and disease similarity network, respectively, to obtain their low dimensional embedding vectors and make predictions through a simple neural network. The feature *x* of each node *v* in these networks is marked as $x_{v},vℬ$ , where ℬ denotes a batch sample set. In each iteration, only the nodes in the batch set are trained. Assuming that the model has *L* layers when sampling the nodes in the batch set, a top–down sampling method is adopted. It collects $n_{k}$ nodes from each layer at a time. Neighborhood sampling functions ${ℋ}_{l}$ of the *l*-*th* layer are defined by sampling the $\;n_{k}$ most similar neighbors of the source node ℬ . ${ℋ}_{l}$ (*v*) represents the sampling set of nodes around the node *v* of the *l*-*th* layer*.* The sampling process is from ${ℬ}^{L}$ to ${ℬ}^{0}$ shown in the sampling section of Algorithm 1. Then we extract the feature $h_{u}^{0}$ of each node *u* in the ${ℬ}^{0}$ set as training features. First, each node v aggregates the representations of the nodes in its sampling neighborhood, $\left\{h_{u}^{l-1},u\in {ℋ}_{l}\left(v\right)\right\}$ into a single vector ${ℋ}_{{ℋ}_{l}\left(v\right)}^{l}$ . After aggregating the neighboring feature vectors, GraphSAGE concatenates the node’s current representation, $h_{v}^{l-1}$ , with the aggregated neighborhood vector, ${ℋ}_{{ℋ}_{l}\left(v\right)}^{l}$ , and this concatenated vector is fed to a fully connected layer with a nonlinear activation function σ , which transforms the representations to be used at the next step of the algorithm for $\;h_{v}^{l}$ . The embedding generation of a given drug node is shown in the embedding section of Algorithm 1. The different aggregator functions can be used in the aggregation steps:

Mean aggregator:

$$\begin{array}{l} h_{{ℋ}_{l}\left(v\right)}^{l}\leftarrow mean\left(\left\{h_{u}^{l-1},\;\forall u\in {ℋ}_{l}\left(v\right)\right\}\right)\\ h_{v}^{l}\leftarrow \sigma \left(W^{l}concat\left(h_{v}^{l-1},h_{{ℋ}_{l}\left(v\right)}^{l}\right)+b^{l}\right). \end{array}$$ (16)

MeanPool aggregator:

$$\begin{array}{l} h_{{ℋ}_{l}\left(v\right)}^{l}\leftarrow mean\left(\left\{\sigma \left(W^{l}h_{u}^{l-1}+b\right),\forall u\in {ℋ}_{l}\left(v\right)\right\}\right)\\ h_{v}^{l}\leftarrow \sigma \left(W^{l}concat\left(h_{v}^{l-1},h_{{ℋ}_{l}\left(v\right)}^{l}\right)+b^{l}\right). \end{array}$$ (17)

MaxPool aggregator:

$$\begin{array}{l} h_{{ℋ}_{l}\left(v\right)}^{l}\leftarrow max\left(\left\{\sigma \left(W^{l}h_{u}^{l-1}+b\right),\forall u\in {ℋ}_{l}\left(v\right)\right\}\right)\\ h_{v}^{l}\leftarrow \sigma \left(W^{l}concat\left(h_{v}^{l-1},h_{{ℋ}_{l}\left(v\right)}^{l}\right)+b^{l}\right). \end{array}$$ (18)

GCN aggregator:

$$h_{v}^{l}\leftarrow \sigma \left(W^{l}mean\left(\left\{h_{v}^{l-1}\right\}\cup \left\{h_{u}^{l-1},\;\forall u\in {ℋ}_{l}\left(v\right)\right\}\right)+b^{l}\right).$$ (19)

LSTM aggregator:

$$\begin{array}{l} h_{{ℋ}_{l}\left(v\right)}^{l}\leftarrow LSTM\left(random\_order\left\{h_{u}^{l-1},\;\forall u\in {ℋ}_{l}\left(v\right)\right\}\right)\\ h_{v}^{l}\leftarrow \sigma \left(W^{l}concat\left(h_{v}^{l-1},h_{{ℋ}_{l}\left(v\right)}^{l}\right)+b^{l}\right), \end{array}$$ (20)

where $W^{l}\;$ and $b^{l}$ are parameter matrix and bias of the *l-th* layer, respectively. The final model outputs a low dimensional embedding vector $z_{v}$ of node *v*. Since formula (19) is a linear approximation of local spectral convolution, it is called a GCN aggregator. It is important to note that LSTM is not inherently symmetric because it processes inputs in a sequential manner. GraphSAGE adopts LSTM to operate on an unordered set by simply applying the LSTM to a random permutation. Unlike GCN, GraphSAGE can perform batch sampling and save the required neighbor features before the node feature aggregation operation. After training, GraphSAGE can perform feature embedding for newly added network nodes. In this way, the network model is actually formed into a subnetwork model according to the sampled nodes, which can increase the learning speed of the model and is suitable for processing larger samples. In this study, the relationship prediction of two types of nodes is involved, and the number of samples is $N_{drug}\times N_{dis}$ , which is very large, so GraphSAGE has better performance. The GraphSAGE minibatch forward propagation is described in Algorithm 1.

Algorithm 1 GraphSAGE minibatch forward propagation in drug–drug interaction or disease similarity network.

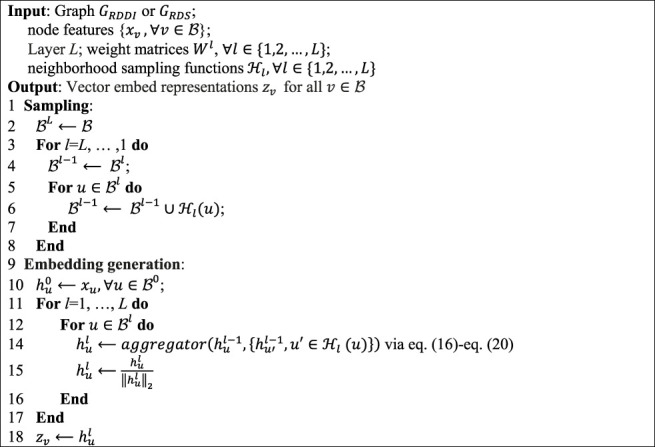

Specifically, we feed the drug attribute feature $F_{drug}$ and drug network clustering feature *U* to the GraphSAGE to get the embedded features $z_{drug}^{F}$ and $z_{drug}^{U}$ and feed the disease attribute feature $F_{dis}$ and disease network feature *V* to the GraphSAGE to get the embedded features $z_{dis}^{F}$ and $z_{dis}^{V}$ . Then we connected the drug embedding features with the disease embedding features to obtain the association features, so as to learn their low dimensional features and predict their relationships. For example, to predict the association between drug *i* and disease *j*, we connect $z_{drug}^{F},\;\;z_{drug}^{U},\;\;z_{dis}^{F}$ and $z_{dis}^{V}$ as *concat* $\left(z_{drug_{i}}^{F},\;z_{drug_{i}}^{U},\;z_{dis_{j}}^{F},\;z_{dis_{j}}^{V}\right)$ , input it into a three-layer fully connected network, and finally use the SoftMax function to find its probability $P_{ij}$ .

### Optimization

GraphSAGE can perform unsupervised learning (Xu et al., 2020), but this objective function is completely based on the topological properties of the network, ignoring the original features of the nodes. If it is applied to this research, each training needs to use a different network. Its essence can reflect the relationship of the features between nodes very well, but it cannot predict the relationship very well. Therefore, we still used the cross-entropy function as the objective function. In order to prevent the over-fitting problem, an L2-regularization is also adopted:

$$Loss=-\sum_{i=1}^{N_{drug}}\sum_{j=1}^{N_{dis}}\left(Y_{ij}logP_{ij}+\left(1-Y_{ij}\right)log\left(1-P_{ij}\right)\right)+\frac{\lambda }{N}\sum_{l=1}^{L}\sum_{w\in W^{l}}w^{2},$$ (21)

where $P_{ij}$ represents the associated probability of drug *i* and disease *j*, $Y_{ij}\in \left\{0,1\right\}$ is the known associations, and $N_{drug}\left(N_{dis}\right)$ is the drug (disease) sample size. Since no negative samples are given in the two databases, extracting reliable negative samples is also an important part of the experiment. The usual operation is to randomly select the same number of negative samples as positive samples from unknown samples. But this will actually interfere with the model learning, so we used the network double random walk (Xie et al., 2012) method to determine the negative samples. After the random walk, the same samples with the smallest scores are regarded as negative samples.

## Experimental Results and Analysis

Based on previous works, we validate our method by answering the following questions:

• Are the features we extracted valid, and can network clustering features improve the performance of the method?
• Can DRGCC predict drug–disease associations with higher accuracy?
• Can we verify that the predicted repositioning drugs are effective, especially for COVID-19?

### Experiment Setting

In our study, we used 5-fold cross-validation (5-fold CV) to evaluate the prediction performance of DRGCC and other competing methods. All samples were randomly divided into five equal-sized parts, four parts of them were used as training data, and the remaining one was used as test data. This process was repeated 5 times, with each part of the data tested once, and the average result of these 5 times was taken as the result of this cross-validation. After that, the samples were randomly divided again, cross-validation was also performed 5 times, and the results were averaged. We mainly used seven metrics: area under the receiver operating characteristic curve (AUC), area under the precision and recall curve (PRAUC), F1_SCORE, ACCURACY, SPECIFICITY, PRECISION, and RECALL (Yu et al., 2020), to comprehensively evaluate the performance of the method. We took the prediction threshold that maximizes the F1_SCORE and built two-layer GraphSAGE models for drugs and diseases separately. After further statistical analysis of drug and disease features, we set some default parameters. The attribute feature dimension $d_{drug}$ of drugs was set to 300, while the attribute feature dimension $d_{dis}$ of diseases was set to 100. The network clustering feature dimension *k* was set to 200. In GraphSAGE, the layer dimensions of drug attribute features were {128, 64}, the layer dimensions of disease attribute features were {64, 32}, and the layer dimensions of network clustering features were {128, 32}. The number of epochs was 30. The learning rate was 0.001. The value of λ in loss function was 0.01. The layer dimensions of a fully connected network were {64, 32, 2}. The dropout was set to 0.5. With the MCODE (Bader and Hogue, 2003) algorithm, the drug and disease (virus) networks in the CTD and HDVD databases were split into 8, 14, and 15, 4 subnetworks, respectively.

### Parameter Sensitivity Analysis

**FIGURE 2:**
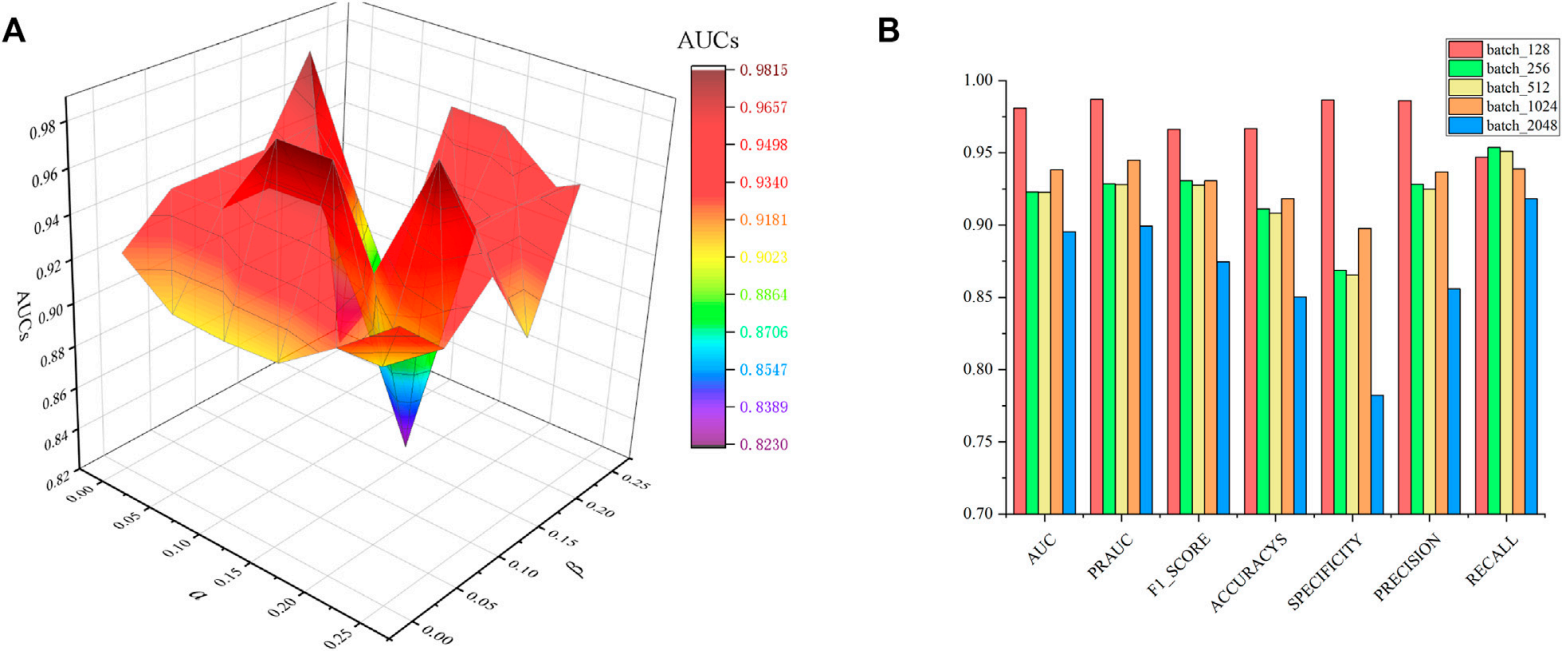
Parametric analysis, **(A)** effects α and β on prediction accuracy, and **(B)** effect of batch size.

In constraint matrix factorization, the regularization of parameters α and β has an important influence on the extraction of network clustering features. We tested all possible combinations of α and β , as shown in Figure 2A. We found that if α = 0.2, β = 0.1, the method has the best AUC value on the CTD dataset. At the same time, since the DRGCC is sampled and trained in batches, the size of the batch is particularly important. If the batch is too small, it will be difficult to converge. If the batch is too large, it demands a large amount of computation. We tested the effect of different batch_size on the method, as shown in Figure 2B. The method has the best performance when the batch_size is equal to 128.

**FIGURE 3:**
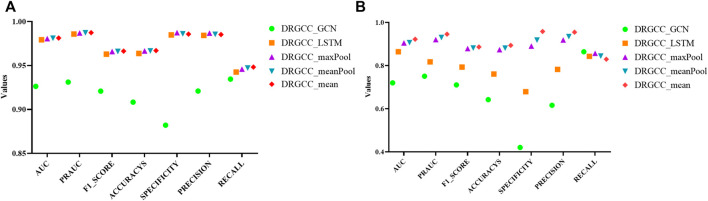
Impact of different aggregation methods on performance. **(A)** Performance of different aggregation methods on the CTD dataset; **(B)** Performance of different aggregation methods on the HDVD dataset.

For the GraphSAGE, there are a total of five different aggregation methods. We performed comparisons on dataset CTD and dataset HDVD, respectively. We can find that the performance of the aggregation methods based on mean, meanPool, and maxPool are similar in Figure 3 and are significantly higher than that of the aggregation based on the LSTM and GCN. This may illustrate that structural features between drugs and symptom features between diseases can be fused using linear methods. Finally, we used the mean method as the aggregation method of the DRGCC.

**FIGURE 4:**
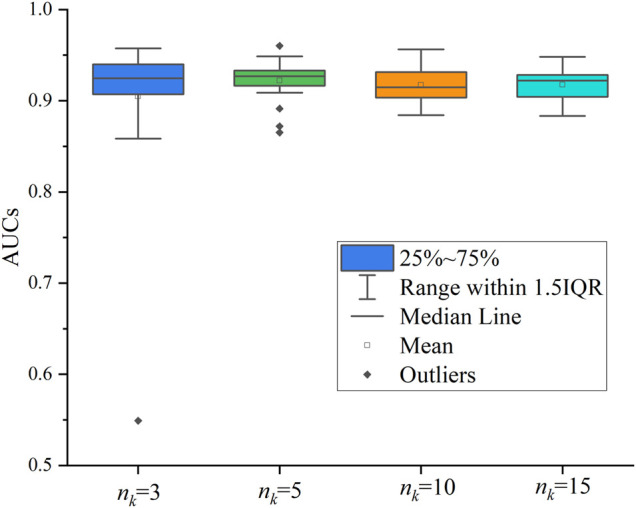
Effect of the number $n_{k}$ of neighbor samples on performance.

We also evaluated the sampling number of network neighbors. Similar to Cui et al. (2021), we tested 4 cases, where $n_{k}$ is {3, 5, 10, 15} and finally determined that it is better to take the nearest 5 neighbor nodes as aggregation nodes. Figure 4 shows the distribution of AUC values for a total of 25 times in 5 cross-validations. This test is run on the HDVD network because it is sparser, and the test on the CTD dataset has a similar effect.

### Effectiveness of Network Clustering Features

**FIGURE 5:**
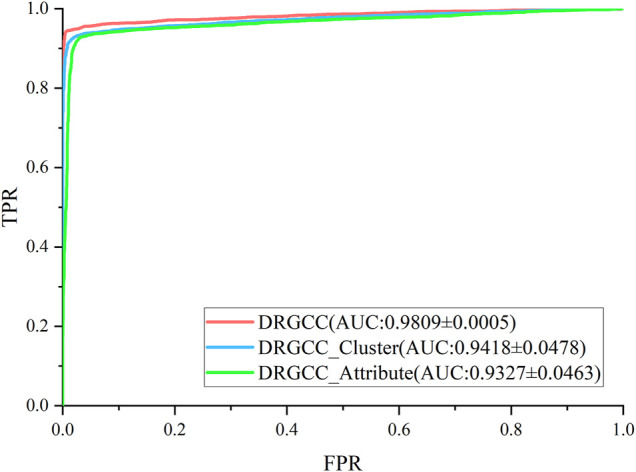
ROC and AUC comparison of DRGCC with different features.

To answer the first question of the experiment, we conduct ablation experiments using only attribute features DRGCC_Attribute and only network clustering features DRGCC_Cluster for prediction, respectively. Table 2 shows that the model with clustering features is slightly higher than the model with only attribute features, and the fusion of the two features has a prominent effect on the CTD database. In Figure 5, the ROC curve of a 5-fold cross-validation is depicted. The average of 5 times is also calculated. It is clear that the performance of applying two features to DRGCC at the same time is better than using a single one, and the AUC is as high as 0.9809. The network clustering feature has a better effect on improving the performance of the method.

**TABLE 2:** Comparison of different features on prediction performance.

Method	AUC	PRAUC	F1_SCORE	ACCURACY	SPECIFICITY	PRECISION	RECALL
DRGCC_Attribute	0.9327 ± 0.0463	0.9379 ± 0.0467	0.9267 ± 0.0278	0.9141 ± 0.0442	0.8893 ± 0.0950	0.9283 ± 0.0459	0.9390 ± 0.0066
DRGCC_Cluster	0.9418 ± 0.0478	0.9477 ± 0.048	0.9430 ± 0.0295	0.9303 ± 0.0459	0.9083 ± 0.0964	0.9477 ± 0.0476	0.9524 ± 0.0050
DRGCC	0.9809 ± 0.0005	0.9871 ± 0.0003	0.9661 ± 0.0006	0.9668 ± 0.0006	0.9866 ± 0.0020	0.9861 ± 0.0020	0.9470 ± 0.0008

### Comparative Analysis With Other Methods

To answer the second question of the experiment, we compared DRGCC with six state-of-the-art drug repositioning methods in this section, such as MBiRW (Luo et al., 2016), DRRS (Luo et al., 2018), BNNR (Zhang W. et al., 2020), SCPMFDR (Meng et al., 2021), NIMCGCN (Li et al., 2020), and LAGCN (Yu et al., 2020) on CTD and HDVD datasets. These methods are mainly divided into three categories: methods based on network propagation, methods based on low-rank matrix approximation, and methods based on the GNN.

• MBiRW (Luo et al., 2016) integrates drug or disease feature information with known drug–disease associations, and the comprehensive similarity measures are developed to calculate similarity for drugs and diseases. They are incorporated into a heterogeneous network with known drug–disease interactions. Based on the drug–disease heterogeneous network, the bi-random walk (BiRW) algorithm is used to identify potential novel indications for a given drug.
• DRRS (Luo et al., 2018) is a matrix completion-based recommendation system on a drug–disease heterogeneous network to predict drug–disease associations.
• BNNR (Zhang W. et al., 2020) is a bounded nuclear norm regularization method to complete a drug–disease heterogeneous network.
• SCPMFDR (Meng et al., 2021) is implemented on an adjacency matrix of a heterogeneous drug–virus network, which integrates the known drug–virus interactions, drug chemical structures, and virus genomic sequences. SCPMF projects the drug–virus interactions matrix into two latent feature matrices for the drugs and viruses, which reconstruct the drug–virus interactions matrix when multiplied together, and then introduces similarity constrained probabilistic matrix factorization to predict associations.
• NIMCGCN (Li et al., 2020) use GCNs to learn latent feature representations of miRNA and disease from the similarity networks and then put the learned features into a neural inductive matrix completion model to obtain a reconstructed association matrix. NIMCGCN is a GCN-based method proposed for the miRNA–disease association prediction, and we adopt it as the baseline method for the drug–disease association.
• LAGCN (Yu et al., 2020) integrates the known drug–disease associations, drug–drug similarities, and disease–disease similarities into a heterogeneous network and applies the graph convolution operation to the network to learn the embeddings of drugs and diseases. It combines the embeddings from multiple graph convolution layers using the attention mechanism.

In Table 3, the results show that our method outperforms other methods on all 7 metrics for the CTD database. In large networks, only considering the relationship between nodes in the network and ignoring the biochemical properties of the nodes themselves have poor prediction performance. For the drug–virus prediction, disease similarity networks based on amino acid sequences and structure-based on drug similarity networks have been provided in HDVD. Since there are no features of viruses in the original dataset, we used DRGCC_cluster for the prediction problem. The prediction results are slightly lower than the results on the CTD due to a large number of unknown relationships and the inclusion of the new virus COVID-19, as shown in Table 4. Except for the RECALL, the other evaluation values are the highest. The AUC reaches 0.9222, and the PRAUC reaches 0.9458. It can be seen that DRGCC has excellent performance.

**TABLE 3:** Performance of comparison methods on CTD dataset.

Method	AUC	PRAUC	F1_SCORE	ACCURACY	SPECIFICITY	PRECISION	RECALL
MbiRW	0.8524 ± 0.0006	0.8487 ± 0.0004	0.7880 ± 0.0016	0.7730 ± 0.0026	0.7025 ± 0.0086	0.7395 ± 0.0047	0.8435 ± 0.0046
DRRS	0.9647 ± 0.0006	0.9655 ± 0.0005	0.9020 ± 0.0009	0.9010 ± 0.0012	0.8909 ± 0.0065	0.8933 ± 0.0053	0.9111 ± 0.0045
BNNR	0.9302 ± 0.0007	0.9479 ± 0.0004	0.8748 ± 0.0012	0.8790 ± 0.0009	0.9120 ± 0.0052	0.9060 ± 0.0045	0.8459 ± 0.0055
SCPMFDR	0.9667 ± 0.0003	0.9734 ± 0.0002	0.9101 ± 0.0011	0.9118 ± 0.0011	0.9304 ± 0.0036	0.9279 ± 0.0032	0.8932 ± 0.0029
NIMCGCN	0.7989 ± 0.0130	0.7311 ± 0.0221	0.8172 ± 0.0081	0.7984 ± 0.0093	0.71780 ± 0.0194	0.7727 ± 0.0173	0.8789 ± 0.0054
LAGCN	0.9259 ± 0.0044	0.7939 ± 0.0054	0.8055 ± 0.0052	0.8843 ± 0.0035	0.8993 ± 0.0091	0.7825 ± 0.0061	0.8314 ± 0.0119
DRGCC	0.9809 ± 0.0005	0.9871 ± 0.0003	0.9661 ± 0.0006	0.9668 ± 0.0006	0.9866 ± 0.0020	0.9861 ± 0.0020	0.9470 ± 0.0008

**TABLE 4:** Performance of comparison methods on HDVD dataset.

Method	AUC	PRAUC	F1_SCORE	ACCURACY	SPECIFICITY	PRECISION	RECALL
MBiRW	0.9113 ± 0.0059	0.9237 ± 0.0052	0.8580 ± 0.0061	0.8541 ± 0.0070	0.8312 ± 0.0149	0.8431 ± 0.0119	0.8769 ± 0.0084
DRRS	0.8936 ± 0.0030	0.92539 ± 0.0021	0.85451 ± 0.0055	0.8664 ± 0.0044	0.9477 ± 0.0117	0.9439 ± 0.0099	0.7851 ± 0.0117
BNNR	0.9088 ± 0.0086	0.93075 ± 0.0062	0.8530 ± 0.0103	0.8580 ± 0.01120	0.8901 ± 0.02428	0.8878 ± 0.0202	0.8260 ± 0.0174
SCPMFDR	0.8655 ± 0.0073	0.8813 ± 0.0064	0.8311 ± 0.0089	0.8325 ± 0.0082	0.84 00± 0.01992	0.8397 ± 0.0141	0.8251 ± 0.0194
NIMCGCN	0.6002 ± 0.0103	0.5922 ± 0.0108	0.7074 ± 0.0034	0.6062 ± 0.0125	0.2686 ± 0.0448	0.5721 ± 0.0167	0.9438 ± 0.0202
LAGCN	0.7433 ± 0.0164	0.5307 ± 0.0097	0.6048 ± 0.0060	0.6989 ± 0.0165	0.6284 ± 0.03212	0.4878 ± 0.0156	0.8105 ± 0.0315
DRGCC	0.9222 ± 0.0080	0.9458 ± 0.0042	0.8863 ± 0.0052	0.8938 ± 0.0048	0.9582 ± 0.0171	0.9548 ± 0.0166	0.8295 ± 0.0165

### Case Studies

To answer the third question of the experiment, we presented an analysis of the predicted repositioned drugs. The top 10 predicted drug–disease relationships were extracted, as shown in Table 5. Among the top 10 prediction results, we can find corroborations or explanations for 6 predictions from other studies. Early evidence in rats suggested that acetazolamide may inhibit sodium and water transport in the ileum in addition to inhibiting bicarbonate secretion (Sladen, 1973). It may have an influence on duodenal ulcer treatment. Rimonabant was shown to be safe and effective in treating the combined cardiovascular risk factors of smoking and obesity (Cleland et al., 2004). Hypoosmolar hyponatremia occurs in conditions of plasma volume depletion such as cirrhosis and heart failure and syndromes of inappropriate antidiuretic hormone secretion. Conventional proposals for euvolemic and hypervolemic hyponatremia consist of lithium carbonate (Gross, 2008). Peyrani *et al.* believed that therapeutics beyond antibiotics (e.g., heparin or aspirin) may be indicated during and after hospitalization for the patients with community-acquired pneumonia (Peyrani and Ramirez 2013). Newer antiemetic with prokinetic properties (cisapride) have also been introduced in the management of gastrointestinal motility disturbances and inflammatory bowel diseases. Some benzodiazepines have been shown to be effective in treating certain anxiety disorders (Swedish Council on Health Technology Assessment, 2005).

**TABLE 5:** Top 10 repositioned drugs predicted by the DRGCC.

Rank	Drug name	Disease name	Evidence (PMID)
1	Acetazolamide	Duodenal ulcer	4360063, Sladen (1973)
2	Salinomycin	Stroke	*NA*
3	Rimonabant	Heart failure	15182777, Cleland *et al.* (2004)
4	Lithium carbonate	Liver cirrhosis and biliary cirrhosis	18480571, Gross (2008)
5	Acetylcarnitine	Hematologic neoplasms	*NA*
6	Heparin	Community-acquired infections	23398875, Peyrani and Ramirez (2013)
7	Icariin	Sialorrhea	*NA*
8	Cisapride	Inflammatory bowel diseases	1974182, Lauritsen *et al.* (1990)
9	Moxifloxacin	Insulin resistance	*NA*
10	Benzodiazepines	Stress disorders, post-traumatic	28876726, Swedish Council on Health Technology Assessment (2005)

The novel coronavirus disease 2019 (COVID-19) pandemic has triggered a massive health crisis and upended economies across the globe. However, the research and development of traditional medicines for the new coronavirus is very expensive in terms of time, manpower, and funds. Drug repurposing emerged as a promising therapeutic strategy during the COVID-19 virus crisis. We also predicted the top 10 possible drugs for anti-COVID-19, as shown in Table 6. Excitingly, seven of them have been reported by medical researchers, such as, triazavirin is a guanine nucleotide analog antiviral that has shown efficacy against influenza A and B, including the H5N1 strain. Given the similarities between SARS-CoV-2 and H5N1, health scientists are investigating triazavirin as an option to combat COVID-19 (Shahab and Sheikhi, 2021) (Valiulin et al., 2021). Aspergillus-producing diseases range from allergic syndromes to chronic lung disease and invasive infections and are frequently observed following COVID-19 infection. Posaconazole has better efficacy with less toxicity for extensive infection and severe immunosuppression (Cadena et al., 2021). In the reports on possible drugs for COVID-19, Uddin *et al.* mentioned that mefloquine may be one of the options (Uddin et al., 2021). In the research of Solanich *et al.*, methylprednisolone and tacrolimus were considered that might be beneficial to treat those COVID-19 patients progressing into severe pulmonary failure and systemic hyperinflammatory syndrome (Solanich et al., 2021). Molnupiravir (EIDD-2801) was originally designed for the treatment of alphavirus infections. Painter *et al.* described its evolution into a potential drug for the prevention and treatment of COVID-19 (Painter et al., 2021). Umifenovir was deemed one of the most hopeful antiviral agents for improving the health of COVID-19 patients (Trivedi et al., 2020). The studies of Lai *et al.* showed that the use of mycophenolic acid might be a strategy to reduce viral replication (Lai et al., 2020).

**TABLE 6:** Top 10 possible anti-COVID-19 drugs predicted by the DRGCC.

Rank	Accession number	Drug name	2D structure	Evidence (PMID)
1	DB15622	Triazavirin	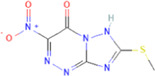	32436829, Shahab and Sheikhi (2021) 33249050, Valiulin *et al.* (2021)
2	DB01263	Posaconazole	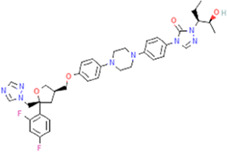	34016284, Cadena *et al.* (2021)
3	DB00358	Mefloquine	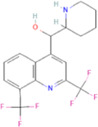	34126913, Uddin *et al.* (2021)
4	DB00864	Tacrolimus	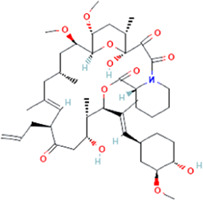	33495742, Solanich *et al.* (2021)
5	DB15661	EIDD-2801	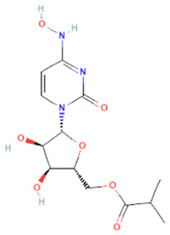	34271264, Painter *et al.* (2021)
6	DB01601	Lopinavir	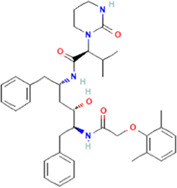	*NA*
7	DB13609	Umifenovir	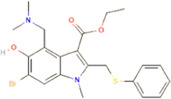	33336780, Trivedi *et al.* (2020)
8	DB11758	Cenicriviroc	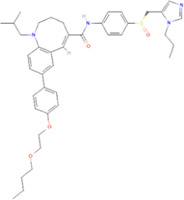	*NA*
9	DB01024	Mycophenolic acid	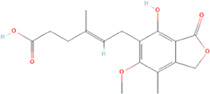	32639598, Lai *et al.* (2020)
10	DB00822	Disulfiram	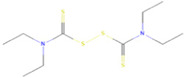	*NA*

**FIGURE 6:**
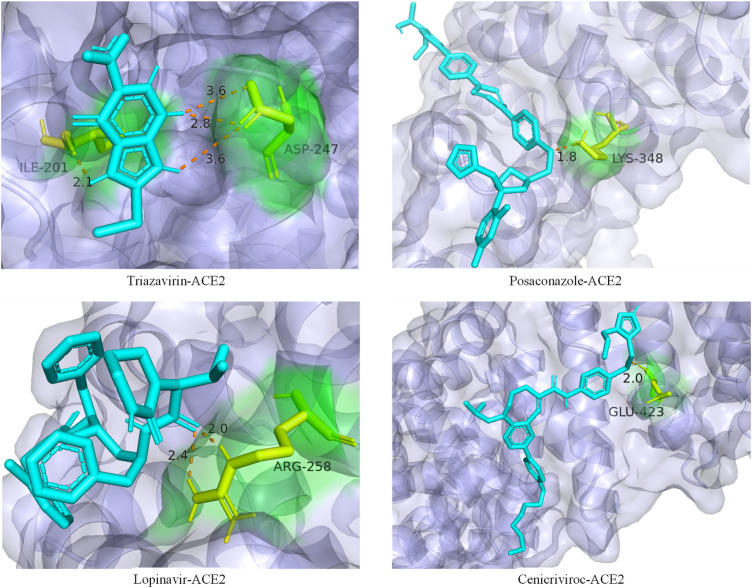
Ligand–protein binding mode between the predicted drugs and the protein receptor ACE2. The purple part is the protein ACE2, the blue part is the drug compound, the yellow part is the amino acid residue, and the orange dotted line is the connecting hydrogen bond. The numbers represent atomic distances.

In addition, we also analyzed the docking state of unverifiable drugs and receptors. Angiotensin-converting enzyme 2 (ACE2) was considered an important functional receptor for SARS and other coronaviruses (Li et al., 2003). Like SARS-CoV, SARS-CoV-2 infects human respiratory epithelial cells through invasion mediated by human cell surface s-protein and ACE2 protein receptors. Obstructing the combination of ACE2 and the virus has become one of the effective means to prevent the respiratory infection of the crown virus. The molecular docking technology allows us to clearly determine the binding sites and bond strengths between molecules (Meng et al., 2011). We examined the binding of 4 drug compounds triazavirin, posaconazole, lopinavir, and cenicriviroc to the receptor protein ACE2. As shown in Figure 6, triazavirin and ACE2 have 4 hydrogen bonds bound to amino acids ILE and ASP, respectively. Lopinavir has 2 hydrogen bonds bound to amino acid ARG in ACE2. Posaconazole and cenicriviroc also have binding sites to ACE2. It can be seen that only one of the 3 unreported drugs has not been corroborated. It can be seen that these drugs may provide some help in the treatment of COVID-19.

## Conclusion

In this article, we have proposed a drug repositioning method DRGCC to predict potential relationships between existing drugs and new diseases. The method first reconstructed the drug–drug interaction network, established the disease semantic similarity network, then extracted the structural features of drugs and disease symptoms as attribute features, and obtained network clustering features through matrix factorization. Finally, all features were fed to the GraphSAGE model to obtain predictions of drug–disease associations. With experiments testing on two datasets, it is found that our method has better performance than other competing methods. Experiments also demonstrated the importance of network clustering features for accurate prediction. At the same time, DRGCC is suitable for training and predicting large-scale samples and can add new nodes to the network after training, such as the SARS-CoV-2 virus. After analyzing the predicted repositioning drugs, we gave several possible drug treatment combinations and recommended several anti-COVID-19 drugs. These predictions have been supported or discussed by other studies. It can be seen that DRGCC has certain reliability in drug repositioning studies.

## Data Availability

Publicly available datasets were analyzed in this study. These data can be found here: http://ctdbase.org/; https://github.com/luckymengmeng/HDVD; https://go.drugbank.com/; https://pubchem.ncbi.nlm.nih.gov/.
